# The neuroleptic drug pimozide inhibits stem-like cell maintenance and tumorigenicity in hepatocellular carcinoma

**DOI:** 10.18632/oncotarget.4307

**Published:** 2015-05-27

**Authors:** Jia-Jie Chen, Nan Cai, Guan-Zhong Chen, Chang-Chang Jia, Dong-Bo Qiu, Cong Du, Wei Liu, Yang Yang, Zi-Jie Long, Qi Zhang

**Affiliations:** ^1^ Organ Transplantation Center, The Third Affiliated Hospital of Sun Yat-Sen University, Guangzhou, Peoples Republic of China; ^2^ Guangdong Provincial Key Laboratory of Liver Disease Research, The Third Affiliated Hospital of Sun Yat-Sen University, Guangzhou, Peoples Republic of China; ^3^ Vaccine Research Institute of Sun Yat-Sen University, The Third Affiliated Hospital of Sun Yat-Sen University, Guangzhou, Peoples Republic of China; ^4^ Department of Hematology, The Third Affiliated Hospital, Sun Yat-Sen University, Guangzhou, Peoples Republic of China

**Keywords:** pimozide, hepatic cancer stem-like cells, self-renewal, STAT3 signaling, STAT3 inhibitor

## Abstract

Drug repurposing is currently an important approach for accelerating drug discovery and development for clinical use. Hepatocellular carcinoma (HCC) presents drug resistance to chemotherapy, and the prognosis is poor due to the existence of liver cancer stem-like cells. In this study, we investigated the effect of the neuroleptic agent pimozide to inhibit stem-like cell maintenance and tumorigenicity in HCC. Our results showed that pimozide functioned as an anti-cancer drug in HCC cells or stem-like cells. Pimozide inhibited cell proliferation and sphere formation capacities in HCC cells by inducing G0/G1 phase cell cycle arrest, as well as inhibited HCC cell migration. Surprisingly, pimozide inhibited the maintenance and tumorigenicity of HCC stem-like cells, particularly the side population (SP) or CD133-positive cells, as evaluated by colony formation, sphere formation and transwell migration assays. Furthermore, pimozide was found to suppress STAT3 activity in HCC cells by attenuating STAT3-dependent luciferase activity and down-regulating the transcription levels of downstream genes of STAT3 signaling. Moreover, pimozide reversed the stem-like cell tumorigenic phenotypes induced by IL-6 treatment in HCC cells. Further, the antitumor effect of pimozide was also proved in the nude mice HCC xenograft model. In short, the anti-psychotic agent pimozide may act as a novel potential anti-tumor agent in treating advanced HCC.

## INTRODUCTION

New advances in drug discovery and development, to make pharmaceutical research more predictable and reliable are urgently needed [1]. One of the most important approaches is drug repurposing, in which new applications for existing or abandoned pharmacotherapies are investigated [2, 3]. In cancer therapy, this technique has achieved affordable results, with new uses found for existing drugs [4]. For example, metformin is the most widely used anti-diabetic drug; however, this drug can also inhibit cancer cell growth *in vitro* and *in vivo*, functioning as an anti-cancer drug [5, 6]. Disulfiram, a drug that is widely used to control alcoholism, also suppresses the self-renewal of glioblastoma and overrides resistance to temozolomide [7]. Furthermore, the use of imatinib (Gleevec), a drug that was originally developed to treat chronic myelogenous leukaemia, has been expanded to treat several malignancies due to its targeting of similar signaling pathways [8], such as those in gastrointestinal stromal tumors [9] and colorectal cancers [10]. Consequently, finding new uses for existing drugs represents an effective strategy for developing novel pharmacotherapies to treat cancer cells.

Hepatocellular carcinoma (HCC) is one of the most common cancers worldwide and is a frequent cause of cancer-related deaths [11]. HCC patients typically have a relatively poor prognosis because most patients are at an advanced stage at the time of diagnosis and few effective therapeutic options are available for this advanced disease. Moreover, HCC cells are often refractory to standard chemotherapy and resistant to radiotherapy [12]. Recurrence or metastasis is quite common in patients, even in those patients undergoing liver cancer resection, and the survival rate is only 30% to 40% at 5 years postoperatively [13]. The existence of tumor-initiating cells or cancer stem-like/stem cells accounts partly for the chemo-resistance observed in HCC [14, 15]. Extensive research over the past decade has identified several specific cellular signaling pathways that are affected in chemo-refractory liver cancer, such as signal transducer and activator of transcription 3 (STAT3), NOTCH, hedgehog and transforming growth factor-beta (TGF-β), which are involved in the self-renewal, differentiation and survival of HCC cells [15–18]. Previous studies have shown that the inhibition of these various signaling pathways via targeted therapy with small molecule drugs appears to be a promising approach for the treatment of HCC [18, 19]. Therefore, the screening of existing drugs that potentially target specific important signaling pathways in HCC is of prime importance for developing novel and effective pharmacotherapies for treating advanced HCC.

Pimozide is an FDA-approved neuroleptic drug that belongs to the diphenylpiperidine class of drugs and that is commonly used to treat Tourette syndrome and schizophrenia [20]. Previous studies have shown that pimozide is efficacious in the treatment of carcinomas and leukaemias, such as melanoma [21], breast cancer [22], chronic myelogenous leukaemia [23] and acute myelogenous leukaemia induced by FLT3 mutations [24]. The neuroleptic agent pimozide inhibited the proliferation of the human breast cancer cell line MCF-7 [22] and enhanced the cell death response of MCF-7 cells to gamma-radiation treatment [25]. Furthermore, pimozide suppressed the self-renewal capacity of chronic myelogenous leukaemia cells by inhibiting the activity of the cellular transcription factor STAT5 [23]. However, the effect of pimozide on HCC cells or stem-like cells and its molecular mechanisms have not yet been fully determined.

The aim of this study was to investigate the inhibiting effects of pimozide on HCC cells and stem-like cells. The results showed that pimozide inhibited cell proliferation, migration, colony formation and sphere formation in HCC cells as well as stem-like cells through suppressing STAT3 activity. To our surprise, pimozide reversed the stem-like cell tumorigenic phenotypes induced by IL-6 addition. Furthermore, pimozide reduced the tumour burden in the nude mice xenograft model. Thus, the anti-psychotic agent pimozide may act as a potential anti-tumor therapeutic drug for HCC treatment, providing a novel therapeutic agent against advanced HCC.

## RESULTS

### Pimozide inhibits HCC cell proliferation in dose- and time-dependent manners by inducing G0/G1 phase cell cycle arrest

**Figure 1:**
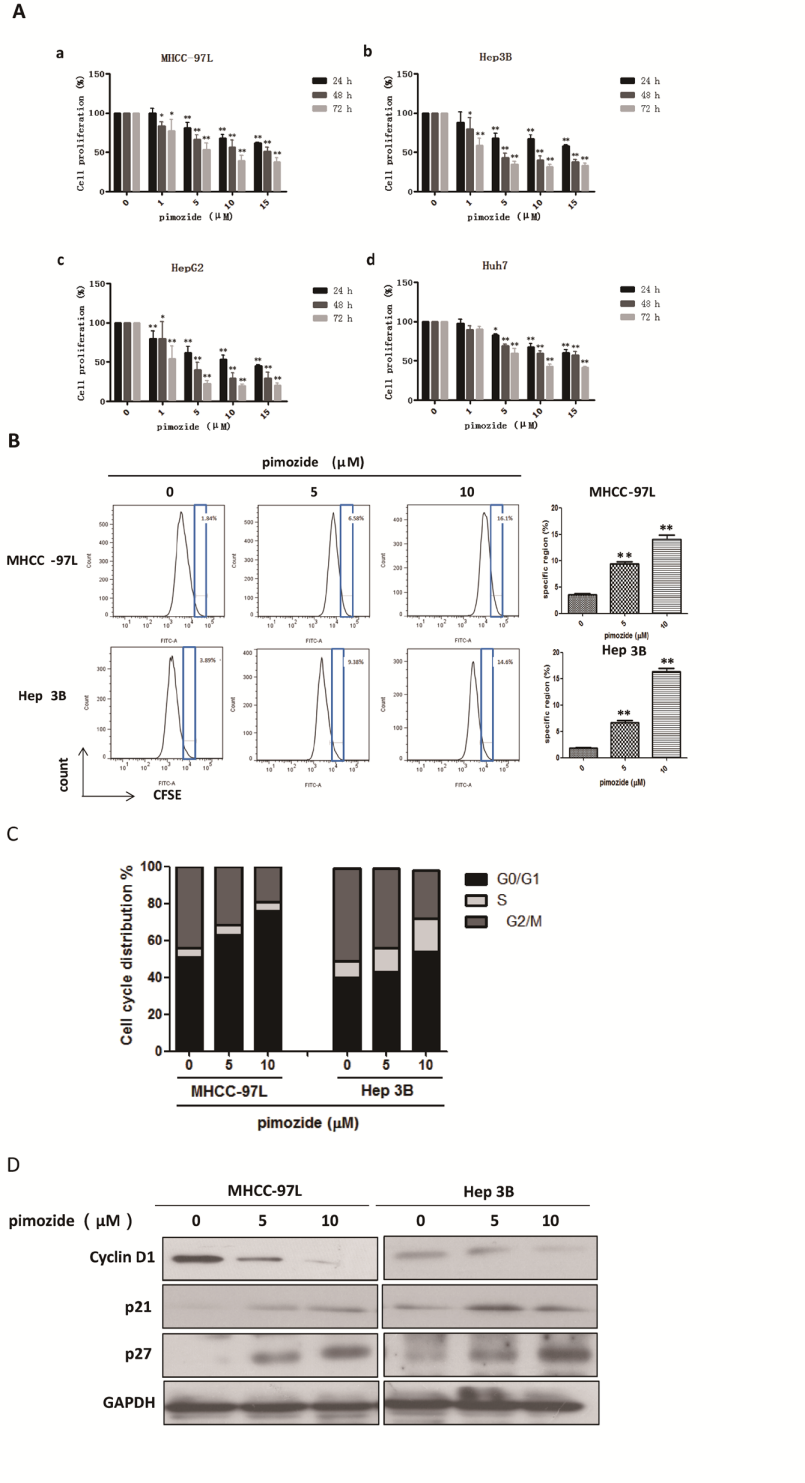
The neuroleptic drug pimozide inhibits HCC cell proliferation in dose- and time-dependent manners by inducing G0/G1 phase cell cycle arrest **A**. MHCC-97L (a), Hep 3B (b), Hep G2 (c) and Huh7 (d) cells were treated with various concentrations of pimozide for various times, and cell viability was determined by MTT assay. **B**. Then, the cells were subjected to flow cytometric analysis to determine the level of CFSE staining. **C**. The cells stained with PI were subjected to flow cytometric analysis to determine the cell distributions at each phase of the cell cycle. The results are shown as the mean values ± SD of 3 independent experiments. *p < 0.05, **p < 0.01, compared with the control. **D**. Western blot analysis of the expression of cell cycle-related genes. Cell extracts were probed with antibodies against p21, p27, Cyclin D1 and GAPDH (loading control) as indicated.

First, the anti-proliferative effect of the neuroleptic drug pimozide in HCC cells was detected using MTT assay. MHCC-97L, Hep 3B, Hep G2 and Huh7 cells were exposed to a series of concentrations (0, 1, 5, 10, and 15 μΜ) of pimozide for 24, 48 and 72 h. As shown in Figure 1A, pimozide inhibited the proliferation of these 4 cell lines in both a dose- and time-dependent manner. The IC50 values at 24, 48 and 72 h were 21.57 ± 3.16, 15.97 ± 0.16 and 6.15 ± 0.48 μΜ for MHCC-97L; 44.37 ± 20.45, 5.29 ± 1.09 and 1.81 ± 0.51 μΜ for Hep 3B; 11.43 ± 0.55, 3.96 ± 0.62 and 1.14 ± 0.27 μΜ for Hep G2; and 20.87 ± 1.54, 20.23 ± 3.31 and 8.44 ± 0.91 μΜ for Huh7 cells, respectively. Next, we examined the effect of pimozide on HCC cells using CFSE staining. The data showed that the specific regions of CFSE staining in MHCC-97L and Hep 3B cells treated with pimozide for 48 h were greater than that of the control (*p* < 0.01) (Figure 1B).

To determine whether pimozide could induce cell cycle arrest, we analysed the effect of pimozide on cell cycle distribution using PI staining. After MHCC-97L and Hep 3B cells were treated with pimozide for 24 h, the percentage of cells in the G0/G1 phase increased significantly compared to the control (*p* < 0.01; Figure 1C). Following treatment with 10 μΜ pimozide, MHCC-97L cells had a significant increase in the percentage of G0/G1 phase cells, from 51.59 ± 3.49% to 76.95 ± 2.98%. Further examination of molecular markers associated with G0/G1 phase arrest showed remarkable increase in the p21 and p27 levels, and a decrease in the cyclin D1 level (Figure 1D), which is consistent with the G1 arrest phenomenon observed by flow cytometric analysis. These results implied that the neuroleptic drug pimozide represented a potential therapeutic index for treating HCC.

### Pimozide inhibits the self-renewal capacity of HCC cells

**Figure 2:**
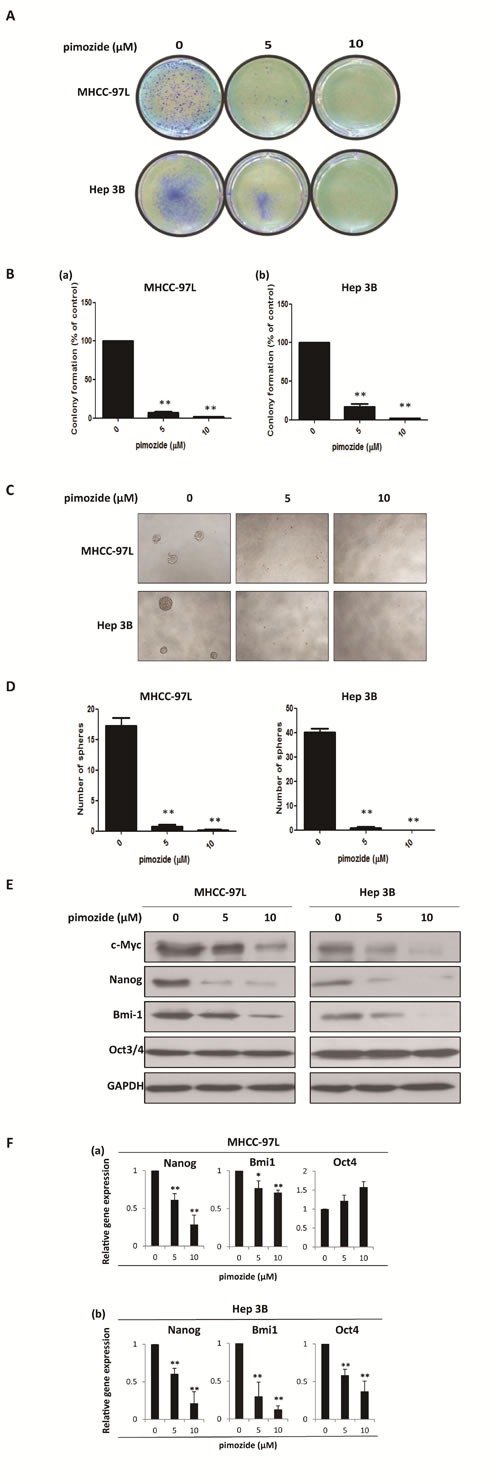
Pimozide inhibits the self-renewal capacity of HCC cells MHCC-97L and Hep 3B cells were treated with pimozide at the indicated concentrations, incubated for extra 10-14 day and then subjected to colony formation assay. Images were taken at a magnification of 100× **A**. The numbers of colonies were counted after staining with crystal violet and the histogram indicated the number of colonies. The results are from 3 independent transfection experiments (**B**). (**C** & **D)**. Sphere formation assay of HCC cells treated with pimozide. The spheres were imaged under a light microscope (magnification, 100× ), and the statistical results are shown. **E**. Western blot analysis of the expression of self-renewal genes. Cell extracts were probed with antibodies against c-Myc, Bmi1, Nanog, Oct3/4 and GAPDH. **F**. MHCC-97L and Hep 3B cells were incubated with the indicated doses of pimozide for 48h before subjected to RT-PCR to detect the expression of the self-renewal genes *Bmi1, Nanog* and *Oct4*. *p < 0.05, **p < 0.01, compared with the control.

Furthermore, we examined whether pimozide inhibited the self-renewal capacity of HCC cells. The colony and sphere formation assays showed that pimozide inhibited the self-renewal capacity of the HCC cell lines MHCC-97L and Hep 3B in a dose-dependent manner (Figure 2A-2D). Following treatment with 5 μΜ pimozide for one week, MHCC-97L cells showed a decrease of 93.0 ± 2.65% in the colony numbers and a significant decrease in the sphere numbers. Similar results were observed in the Hep 3B cells.

The expression levels of self-renewal-related proteins were measured by western blot analysis to delineate the mechanism of pimozide activity (Figure 2E). HCC cells treated with pimozide for 48h demonstrated significantly down-regulated expression of stemness protein, including Bmi-1, c-Myc and Nanog, but showed no significant change in OCT3/4 expression. In addition, RT-PCR assay showed that pimozide down-regulated transcriptional expression levels of *Bmi1* and *Nanog* of HCC cells in a dose-dependent manner (Figure 2F). These results indicated that pimozide inhibited the self-renewal capacity of HCC cells by suppressing the expression of key stemness transcription factors Bmi-1, c-Myc and Nanog.

### Pimozide suppresses HCC cell migration

**Figure 3:**
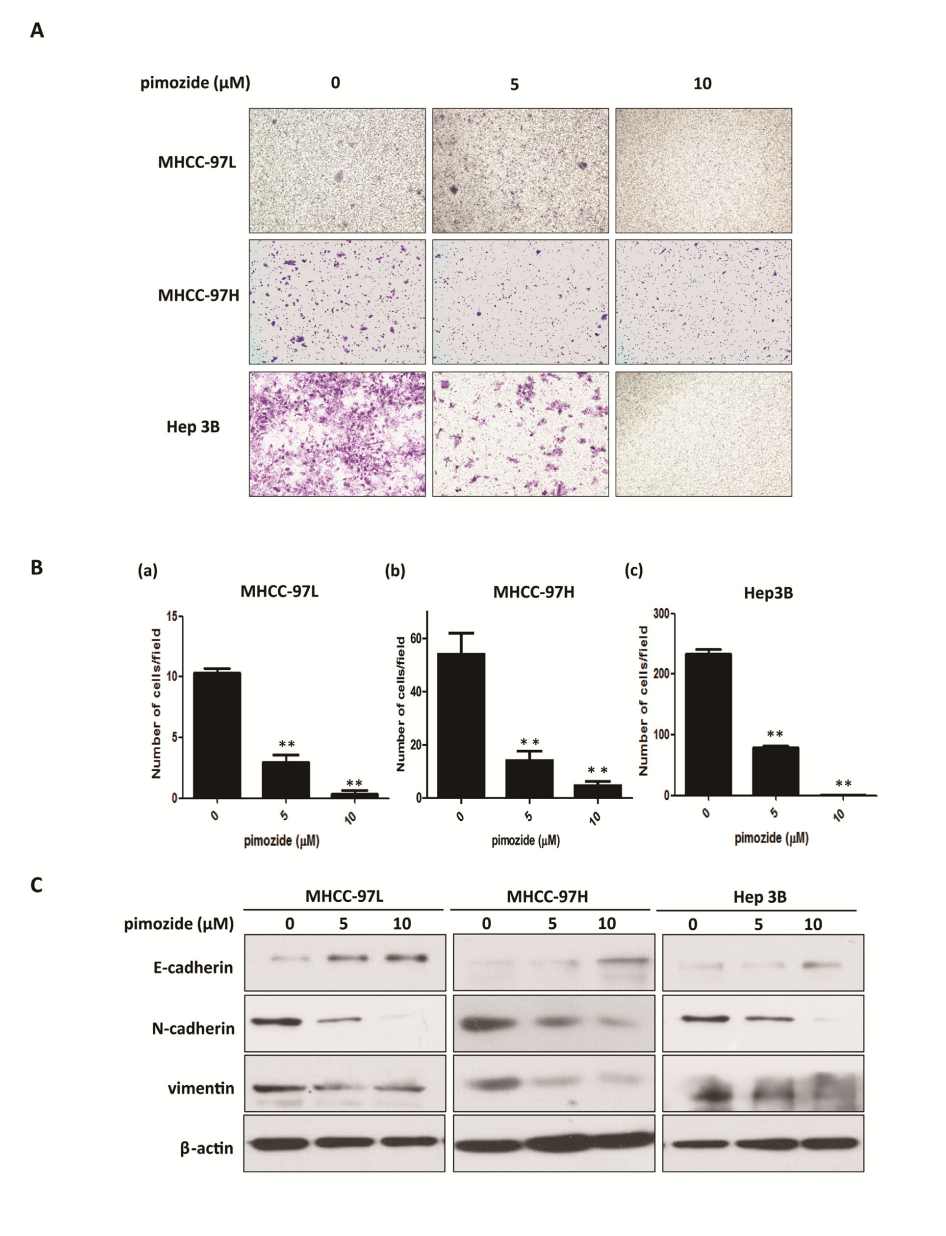
Pimozide suppresses HCC cell migration Transwell migration assays of HCC cells treated with pimozide for 48 h. **A**. Images shown at a magnification of 100×. **B**. The data are summarized from 3 independent experiments, *p < 0.05, **p < 0.01, compared to the control. **C**. Western blotting analysis of the expression of EMT-related markers. Cell extracts were probed with antibodies directed against E-cadherin, N-cadherin, vimentin and β-actin (loading control) as indicated.

As shown in Figure 3A and 3B, all of MHCC-97L, MHCC-97H and Hep 3B cells demonstrated reduced cell migration capacity after treatment with 5 and 10 μΜ pimozide compared to the control, as evaluated by transwell migration assay. The ability to migrate in chambers without a matrix was significantly reduced to 29.03 ± 9.68% and 25.80 ± 5.07% respectively in MHCC-97L and MHCC-97H cells, after 5 μΜ pimozide treatment (Figure 3A and 3B). Furthermore, Hep 3B cells treated with 5 μΜ pimozide had a decrease of 66.5 ± 2.43% of migrated cells compared to the control (*p* < 0.01; Figure 3A and 3B).

In order to detect the toxicity or side effects of pimozide, apoptotic cell percentage was measured by Annexin-V-FITC/PI double staining assay. The result showed that pimozide did not induce obvious apoptosis of HCC cells (Supplemental Figure 2A and 2B). Moreover, epithelial-mesenchymal transition (EMT) has gained increasing attention due to its importance in the acquisition of metastatic and drug-resistant potential during tumour progression [28]. Here, we assessed whether pimozide inhibited HCC cell migration via the EMT process. Western blot analysis was performed to validate the expression of the EMT markers E-cadherin, N-cadherin and vimentin. Pimozide down-regulated the expression levels of N-cadherin and vimentin and up-regulated the expression level of E-cadherin in a dose-dependent manner, suggesting that pimozide inhibited HCC cell migration by suppressing EMT markers expression (Figure 3C).

### Pimozide inhibits the self-renewal and migration capacities of SP HCC cells

**Figure 4:**
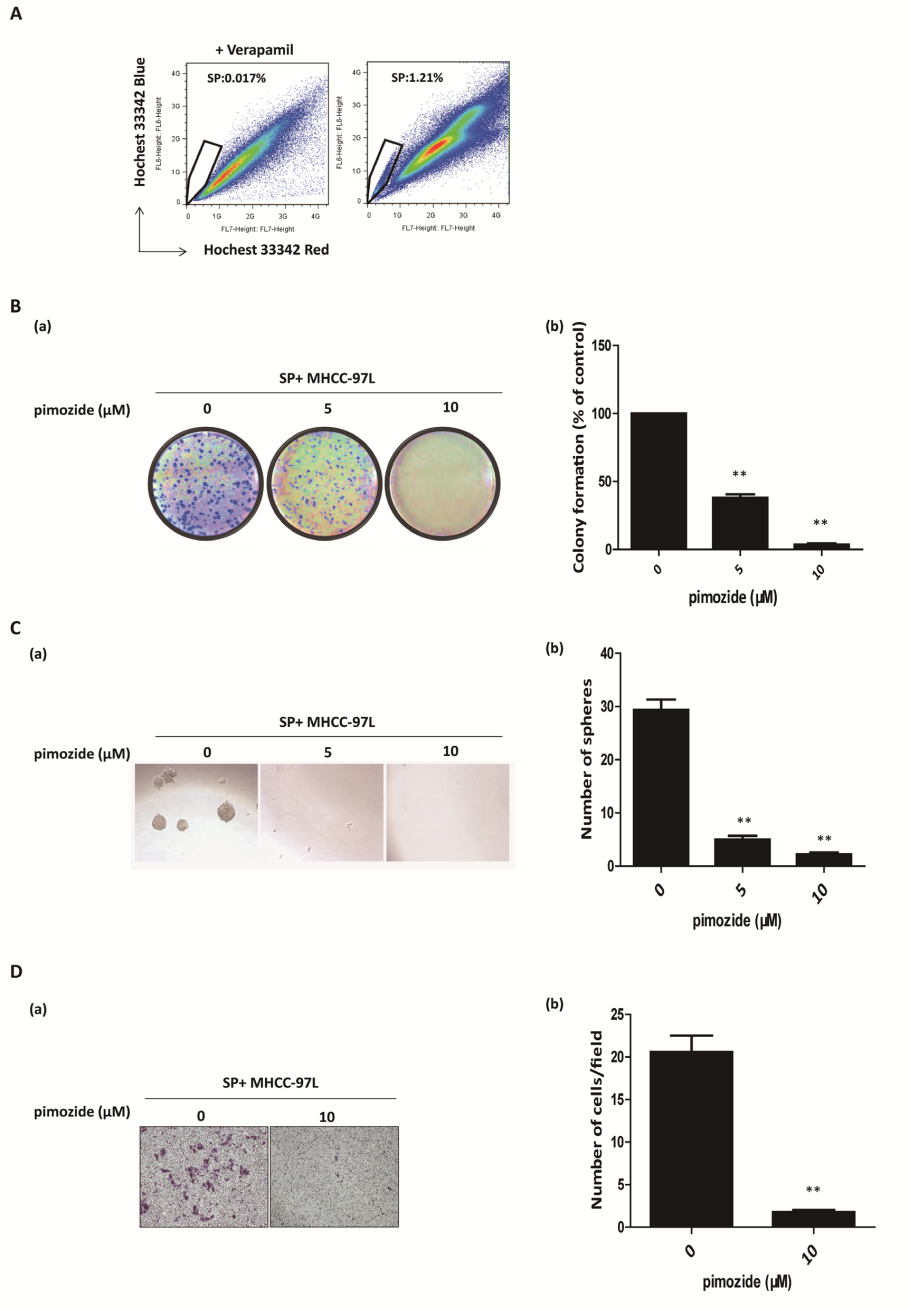
Pimozide inhibits the self-renewal and migration capacities of SP HCC cells **A**. Fluorescence-activated cell sorting isolation of SP MHCC-97L cells. **B**. Colony formation assay of SP MHCC-97L cells treated with 5μM or 10μM pimozide. The images (a) and statistical results (b) are shown. **C**. Sphere formation assay of SP MHCC-97L cells treated with pimozide. **D**. Transwell migration assay of SP MHCC-97L cells treated with 10 μM pimozide for 48h. Images are shown at a magnification of 100×, and the statistical results are shown. The data are summarized from 3 independent experiments, *p < 0.05, **p < 0.01, compared to the control.

Cancer stem and stem-like cells play a pivotal role in carcinogenesis and tumour recurrence. A previous study showed that SP cells were a minor subset of cancer stem-like cells in the HCC cell line MHCC-97L [29]. To clarify the effect of pimozide on HCC stem-like cells, the SP fraction in MHCC-97L cells was sorted by flow cytometric analysis (Figure 4A) and displayed respectively resistant to fluorouracil (5-FU), cisplatin (Cis) and doxorubicin (Dox) (Supplemental Figure 1A). Pimozide suppressed the colony and sphere formation capacities in these SP cells compared to the control (Figure 4B and 4C). MHCC-97L SP cells showed a significant 62% reduction in colony numbers and an 83% decrease in sphere numbers after treatment with 5 μM pimozide. Figure 4D showed that pimozide treatment inhibited SP cell migration, as evaluated by transwell migration assay. These data suggested that pimozide has the potential to inhibit the self-renewal and migration capacities of HCC SP cells *in vitro*.

### Pimozide inhibits the self-renewal and migration capacities of CD133-positive HCC cells

**Figure 5:**
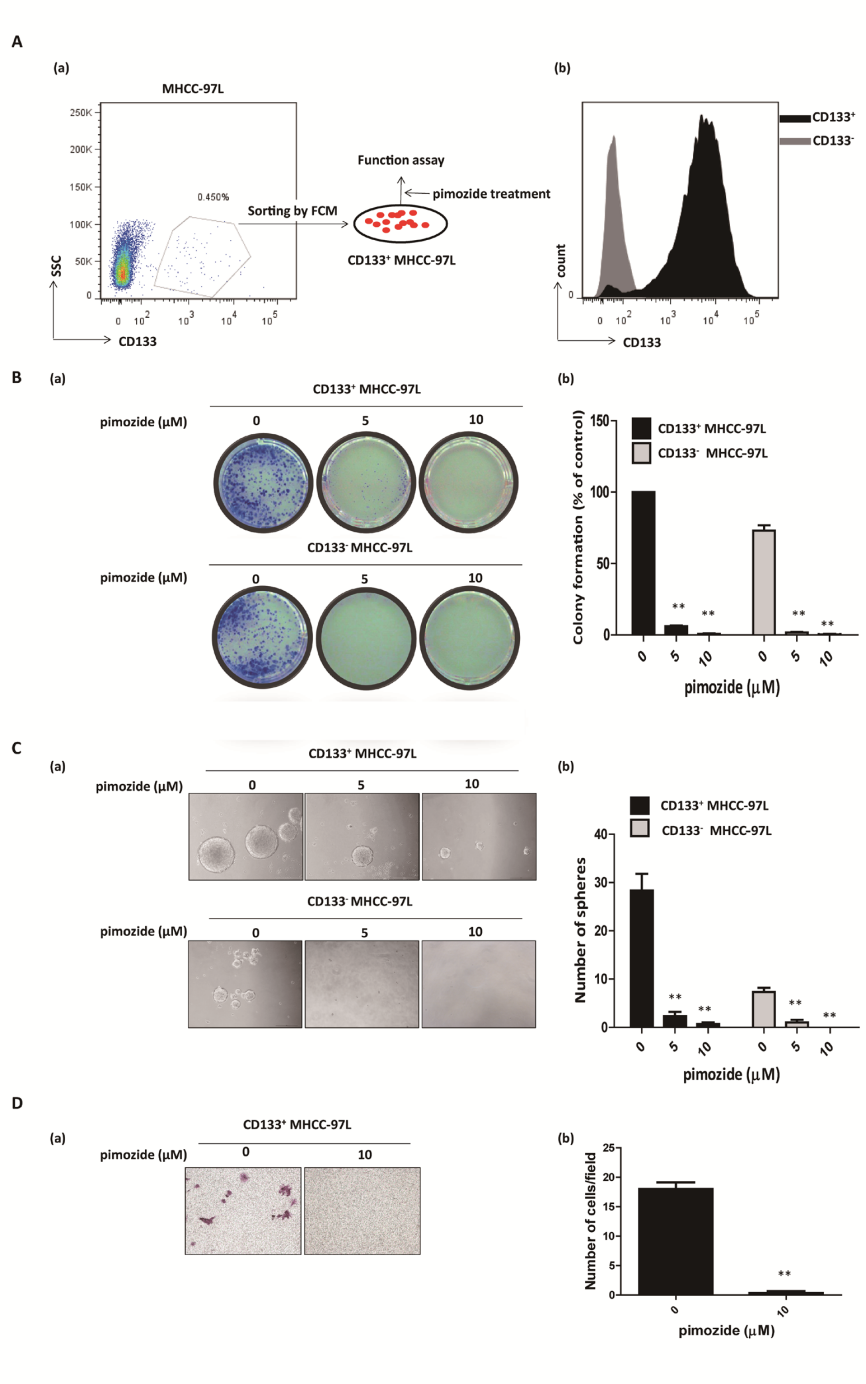
Pimozide inhibits the self-renewal and migration capacities of CD133-positive HCC cells **A**. Fluorescence-activated cell sorting isolation of CD133-positive cells and negative cells from MHCC-97L cells (a). The purity of fluorescence-activated cell sorting isolation was confirmed (b). **B**. Colony formation assay of CD133-positive and negative MHCC-97L cells treated with the indicated doses of pimozide. The images (a) and statistical results (b) are shown. **C**. Sphere formation assay of CD133-positive and negative MHCC-97L cells treated with pimozide. **D**. Transwell migration assay of CD133-positive MHCC-97L cells treated with 10 μM pimozide for 24h. Images are shown at a magnification of 100×, and the statistical results are shown. The data are summarized from 3 independent experiments, *p < 0.05, **p < 0.01, compared to the control.

CD133 is a candidate marker for enriching cancer stem or progenitor cells in HCC [30]. Pimozide obviously reduced CD133-positive populations in MHCC-97L cell lines at the concentration of 10 μΜ (Supplemental Figure 3). Also, we further tested whether pimozide showed an anti-proliferative effect on CD133-positive MHCC-97L cells. As shown in Figure 5A and 5B, CD133-positive fraction was obtained by fluorescence-activated cell sorting, and showed resistance to 5-FU, Cis and Dox (Supplemental Figure 1B). Colony and sphere formation assays showed that pimozide inhibited the self-renewal capacity of CD133-positive MHCC-97L cells (Figure 5B and 5C). Following treatment with 5 μΜ pimozide for one week, CD133-positive cells showed a decrease of 94.0 ± 1.0% in colony numbers and a significant decrease in sphere numbers. Similar results were shown in CD133-negative cells. Furthermore, CD133-positive cells treated with 10 μΜ pimozide had a significant decrease in migrated cells compared to the control (*p* < 0.01; Figure 5D). These results indicated that pimozide could inhibit the self-renewal and migration capacities of CD133-positive HCC cells *in vitro*.

### Pimozide suppresses STAT3 signaling and reverses cancer stem-like cell phenotypes induced by IL-6 addition in HCC cells

Dysregulated STAT3 signalling has been strongly implicated in tumourigenesis through its effects on cell growth, angiogenesis, immune system evasion, and apoptosis prevention [31]. The activation of STAT3 signaling was reported to maintain the tumor-initiating ability in HCC cells, with high expression of STAT3 phosphorylation at tyrosine 705 [32]. The STAT3 phosphorylation in CD133-positive HCC cells or SP HCC cells were detected using Western blotting assay. Our results showed that CD133-positive cells or SP cells from MHCC-97L cells had high expression of phosphorylated STAT3 (Tyr705) and downstream gene c-Myc (Supplemental Figure 1C), indicating that in stem-like cells of HCC STAT3 signalling was fully activated.

**Figure 6:**
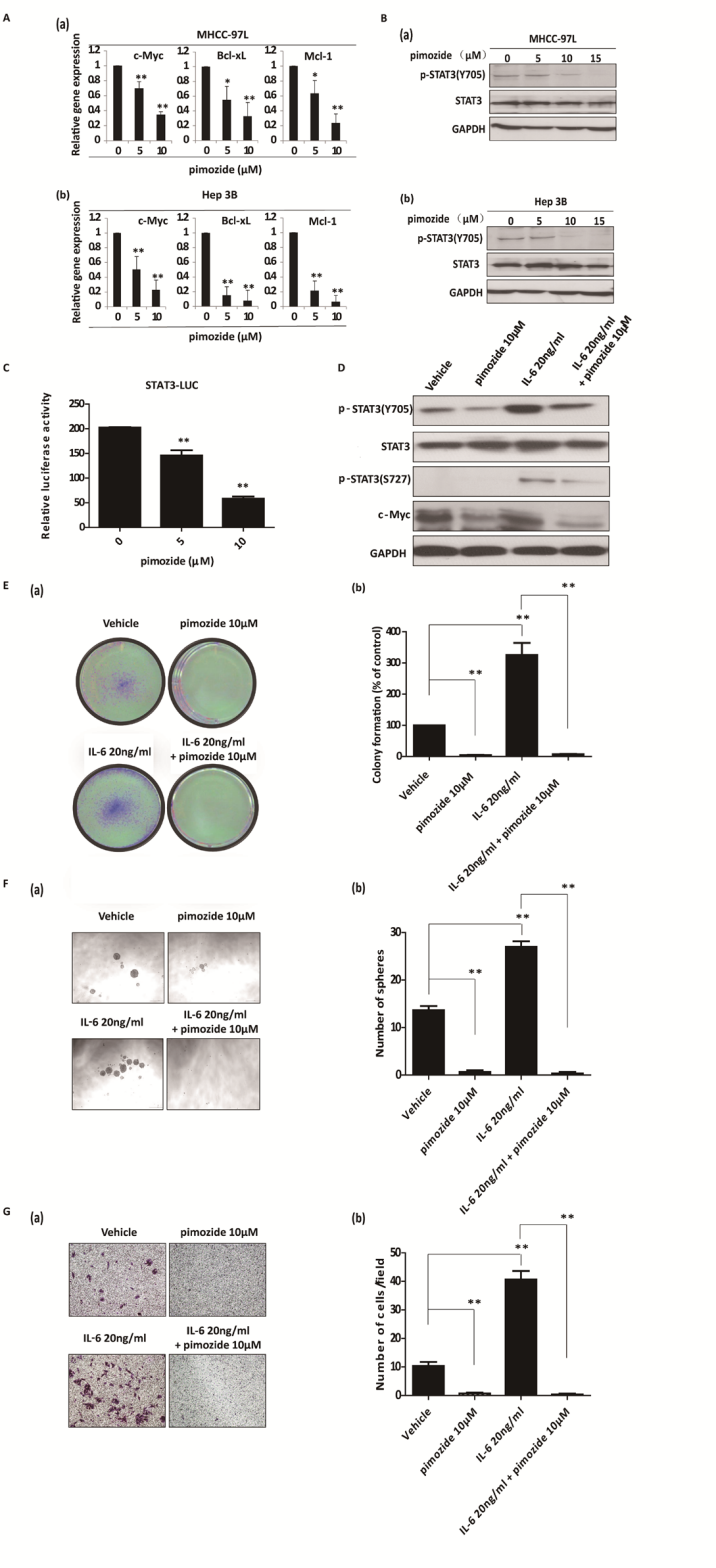
Pimozide suppresses the activity of STAT3 signaling and reverses cancer stem-like cell phenotypes induced by IL-6 addition in HCC cells **A**. MHCC-97L and Hep 3B cells were incubated with the different doses of pimozide for 48h before subjected to RT-PCR to analyse the expression of the STAT3 downstream genes *c-Myc*, *Bcl-xL* and *Mcl-1*. **B**. Western blot analysis of the expression of phosphorylated STAT3 (p-STAT3 Y705), STAT3 and c-Myc. **C**. HEK293T cells were transfected with STAT3 reporter and pRL-TK renilla luciferase reporter plasmids. After 24 hours of transfection, the cells were treated with different doses of pimozide for another 48h. Dual luciferase assay was performed to detect the relative luciferase activity. MHCC-97L cells were treated with 20 ng/ml IL-6, 10 μM pimozide or a combination as indicated for 12 h and subjected to western blot analysis (**D**), colony (**E**) and sphere (**F**) formation assays, and transwell migration assay (**G**) Relative representative figures are shown. The data are summarized from 3 independent experiments, *p < 0.05, **p < 0.01.

Using qRT-PCR, we found that pimozide treatment inhibited the transcriptional levels of the STAT3 signaling related downstream genes *c-Myc*, *Bcl-xL* and *Mcl-1* (Figure 6A). In addition, pimozide reduced phosphorylation of STAT3 (Tyr705) expression in both MHCC-97L and Hep 3B cells in a dose-dependent manner (Figure 6B). Furthermore, pimozide inhibited STAT3-dependent luciferase activity in a dose-dependent manner (Figure 6C). IL-6 exerts many functions via the activation of STAT3 signaling in HCC cells, showing high expression of STAT3 phosphorylation at tyrosine 705 and at serine 727 [33]. Western blot analysis showed that IL-6 activated STAT3 signaling in MHCC-97L cells with high level of phosphorylated STAT3 (Y705), phosphorylated STAT3 (S727) and c-Myc expression (Figure 6D). However, pimozide treatment rescued the phosphorylation expression of STAT3 at the sites of both Tyr 705 and Ser 727, as well as reduced the downstream c-Myc expression induced by IL-6 addition (Figure 6D).

Moreover, we examined whether pimozide reversed the phenotypes of cancer stem-like cells induced by IL-6 treatment in HCC cells. Functional assays showed that IL-6 treatment significantly enhanced the self-renewal and migration capacities, as evaluated by *in vitro* colony formation, sphere formation and transwell migration assays in MHCC-97L cells, suggesting that IL-6 addition could enhance the stemness of HCC cells (Figure 6D-6G). In contrast, pimozide could reverse the IL-6-induced self-renewal and migration capacities of these cells (Figure 6D-6G), further suggesting that pimozide inhibited STAT3 signaling activity to suppress stem-like cell maintenance and tumorigenicity in HCC cells.

### Pimozide reduces the tumour burden in a nude mouse HCC-xenograft model

**Figure 7:**
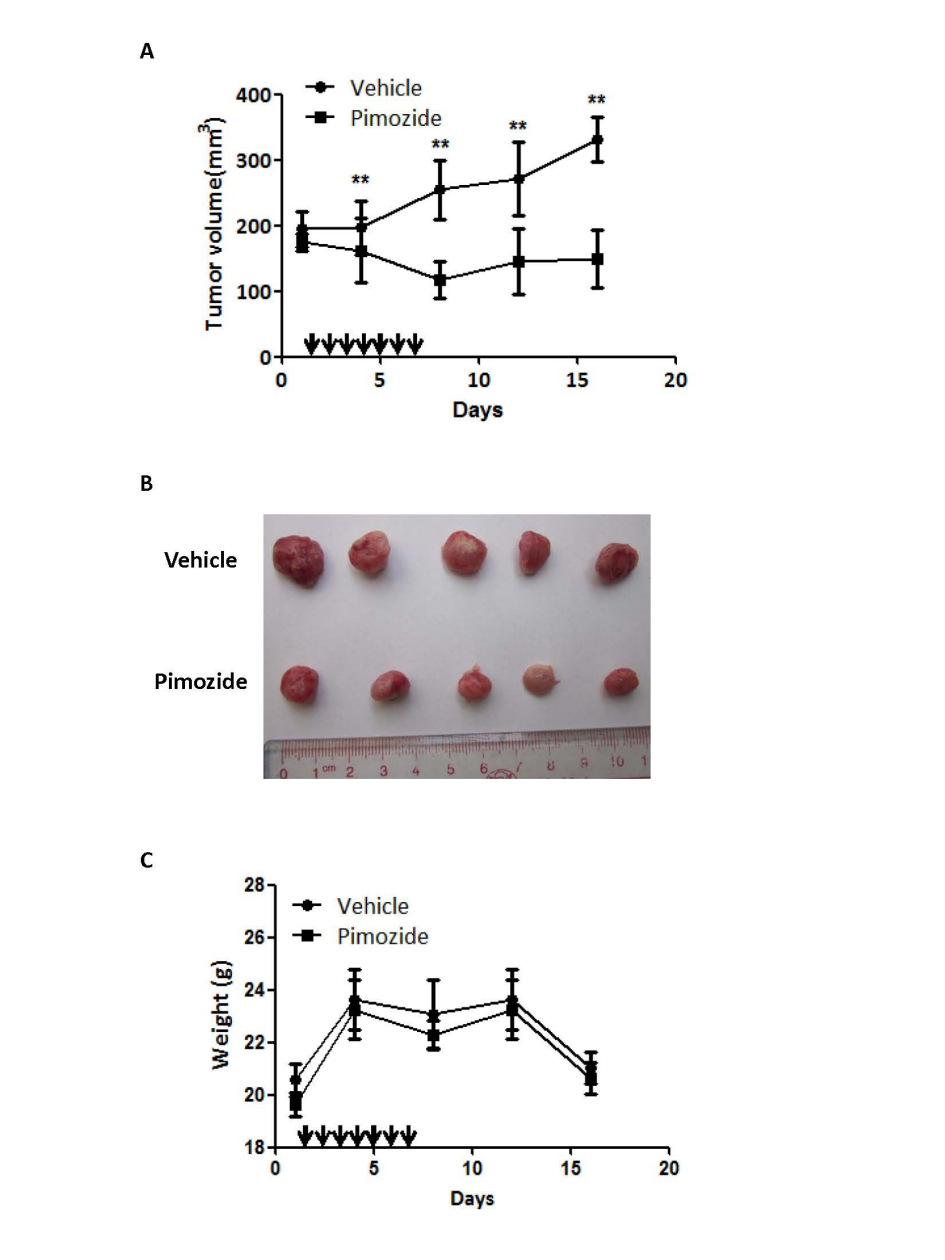
Pimozide reduces the tumor burden in a nude mice xenograft model Pimozide was administered to mice previously injected with MHCC-97L cells. **A**. The tumour volume was measured and analysed at the indicated time points. Points, mean; bars, SE, **p < 0.01. **B**. Representative tumors removed from mice of each group are shown. **C**. Body weights were determined for the animals in each treatment group. Points, mean; bars, SE.

Since pimozide reduced cell viability by inhibiting proliferation and self-renewal in cellular models, we next examined the effect of pimozide in a nude mice xenograft model. MHCC-97L hepatic cancer cells were injected subcutaneously into immunodeficient nude mice. Treatment with pimozide reduced the tumor burden to a level comparable to that of the control group, as assessed by tumor volume (Figure 7A and 7B). Furthermore, both treatments were well tolerated, with no significant effects on body weight (Figure 7C). These data indicated that the neuroleptic drug pimozide had anti-tumor efficacy *in vivo* without overt toxicity.

## DISCUSSION

In recent years, increasing numbers of biotechnology companies have focused on drug repurposing, which is the development of novel uses for existing drugs [34]. Many existing drugs, such as metformin and disulfiram, have demonstrated anti-cancer effects in addition to their original uses. Compared with other types of cancer, HCC may benefit more from drug repurposing due to its poor prognosis and lack of effective chemotherapeutic agents [35].

Pimozide is an FDA-approved neuroleptic drug in clinical use. Due to its advantages of relatively low side effects and high clinical safety, pimozide has been used to treat other diseases in addition to central nervous system (CNS) diseases. During the past 20 years, pimozide has been found to be efficacious in treating monosymptomatic hypochondriacal psychoses, body dysmorphic disorder, metastatic melanoma, trichotillomania, as well as trigeminal and postherpetic neuralgia [36]. This study is the first to report that pimozide could be used as a new anti-cancer drug for treating HCC.

In our present study, we found that pimozide inhibited liver cancer cell proliferation and sphere formation by inducing G0/G1 phase cell cycle arrest. Previous studies have demonstrated that HCC cancer stem/stem-like cells are not targeted by current systemic chemotherapies and appear to be resistant to the conventional chemotherapeutic agents Cis, 5-FU and Dox [28, 37]. For example, the SP fraction of the HCC cell line MHCC-97L was found to be refractory to the drug DOX [28]. Furthermore, CD133-positive hepatic cancer cells are resistant to conventional chemotherapeutic agents (5-FU and DOX) [38]. In this study, MTT assay showed that CD133 positive cells or SP cells form MHCC-97L were respectively resistant to 5-FU, Cis and Dox, compared with their control cells (Supplemental Figure 1A & 1B). To our surprise, we found that pimozide significantly inhibited the colony formation,sphere formation and cell migration abilities of SP or CD133-positive HCC cells. Down-regulated expression of the stemness genes Bmi-1, c-Myc and Nanog was also detected, which indicated that pimozide inhibited the self-renewal capacity of HCC cancer stem-like cells and might be preferable to conventional chemotherapy for targeting liver cancer stem/stem-like cells.

To delineate the underlying mechanism of the anti-cancer effect of pimozide on HCC cells, we tested select classical pathways involved in HCC carcinogenesis and found that the STAT3 pathway was significantly suppressed after pimozide treatment. As an oncogene, STAT3 is constitutively activated by the phosphorylation of Tyr705 in various human malignancies [31]. STAT3 is involved in oncogenesis, cell proliferation, angiogenesis, self-renewal and drug resistance. Phosphorylated STAT3 dimerizes and translocates into the nucleus for binding to specific DNA response elements to induce the transcription of downstream genes, such as *BCL-xL*, *MCL1*, and *c-Myc* [39]. Accumulating evidence has shown that of the inhibition of STAT3 activity in cancer cells can inhibit tumor growth and enhance chemotherapy sensitivity in HCC cells [40, 41]. In addition, several clinical studies have reported that STAT3 signaling activation is associated with poor prognosis in HCC patients [42, 43]. Thus, targeting the STAT3 pathway using specific inhibitors may be a useful cancer treatment strategy with the potential for broad clinical application. The Janus kinase 2 inhibitor AG490 can abrogate the activation of constitutive STAT3 to inhibit growth and metastasis, and overcome drug resistance in human HCC cells [44]. In addition, sorafenib, a first-line therapy in treating patients with advanced liver cancer, demonstrats anti-tumor activity through the inhibition of STAT3 signaling [45]. Several other chemicals have also shown anti-cancer activity in treating HCC cells through the inhibition of STAT3 signaling [46, 47]. In the present study, pimozide inhibited STAT3 phosphorylation and STAT3-dependent luciferase activity, and down-regulated the transcription levels of STAT3 signaling downstream genes *BCL-xL*, *MCL1*, and *c-Myc*, significantly indicating that pimozide targeted STAT3 signaling.

IL-6 induces STAT3 phosphorylation at Tyr-705 and Ser-727 through IL-6 receptors and JAK to activate STAT3 signalling in HCC cells. IL-6/STAT3 signaling promotes survival, anti-apoptosis, migration, drug resistance and self-renewal [48]. Likewise, our data showed that pimozide decreased IL6-mediated STAT3 phosphorylation and could reverse cancer stem-like cell phenotypes induced by IL-6 addition in HCC cells, further suggesting that pimozide may be a potential agent for treating HCC cells by inhibiting STAT3 signaling and suppressing cancer stem-like cell maintenance. Besides, since pimozide is a well-known antagonist of serotonin 5-hydroxytryptamine receptor 7 (5HT7), which was overexpressed in human hepatocellular cancer (Data not shown, [49]), whether 5HT7 expression is associated with STAT3 activation in HCC cells is required to investigated in future.

Although pimozide causes cardiac toxicity, it has not been shown to have adverse effects on other normal functional cells, such as hepatic or haematopoietic cells. A previous study shows that pimozide decreases the colony formation ability of bone marrow progenitor cells derived from patients with CML. However, pimozide has almost no effect on haematopoietic progenitors derived from healthy donors [24]. Furthermore, pimozide treatments are well tolerated, with no significant effects on body weight in a mouse model [24]. Similar results were observed in our study. Additionally, according to the previous study, the precise lethal dose of pimozide in humans is unknown. The oral LD50 is 228 mg/kg in mice, 5120 mg/kg in rats, 188 mg/kg in guinea pigs, and 40 mg/kg in dogs (DrugBank: Pimozide (DB01100) [50]). The dose of pimozide (25 mg/kg) used in our research is relatively lower compared to the commonly used dose for treating CNS disease. Therefore, pimozide may also be a safe drug for treating HCC.

In conclusion, this study demonstrates that the neuroleptic drug pimozide displays anti-tumour activity against HCC cells or stem-like cells and may be a novel candidate drug for treating advanced HCC. Of note, the development of new pimozide derivatives with stronger anti-cancer effects but lower side effects are urgently needed for advanced HCC [51].

## MATERIALS AND METHODS

### Cell lines and cell culture

The human HCC cell lines MHCC-97L and MHCC-97H (purchased from the Liver Cancer Institute, Zhongshan Hospital, Fudan University, Shanghai, China), HEK293T, Huh7 (provided by the Shanghai Cell Collection, Chinese Academy of Sciences, Shanghai, China), Hep 3B, and Hep G2 (purchased from American Type Culture Collection, Manassas, VA, USA) were cultured in Dulbecco's modified Eagle's medium (DMEM; Gibco, Grand Island, NY, USA) containing 10% fetal bovine serum (FBS; Sigma, St. Louis, MO, USA). All media were supplemented with 100 units/ml penicillin and 100 μg/ml streptomycin. All cell lines were kept at 37°C in a 5% CO_2_ humidified atmosphere.

### Cell proliferation assay using MTT and CFSE staining

Cell proliferation was evaluated by MTT assay according to the manufacturer's instructions. The human HCC cell lines MHCC-97L, Hep 3B, Hep G2 and Huh7 were seeded in 96-well culture plates with 2,000 cells per well. Subsequently, the cells were treated with pimozide at different concentrations for various time intervals (24 h, 48 h and 72 h). The cells were incubated for another 4 h at 37°C after 20 μL MTT (5 mg/ml) solution was added to each well. The supernatant fluid was removed, and 150 μL DMSO was added to each well. The absorbance at 490 nm was finally measured using a microplate reader (BioTek, Vermont, USA).

To provide a direct measure of cell proliferation, 5- or 6-(N-succinimidyloxycarbonyl)-3,6-O,O-diacetylfluorescein (CFSE, Sigma, St. Louis, MO, USA) staining was also used for flow cytometric analysis of cells treated with pimozide for 48 h according to the manufacturer's instructions.

### Cell cycle assay

Cell cycle distribution was determined by propidium iodide (PI, Sigma, St. Louis, MO, USA) staining and flow cytometric analysis according to the manufacturer's instructions. Briefly, equal amounts of cells were seeded in 6-well plates and treated with pimozide at different concentrations for 48 h. After suspended in cold absolute ethanol, the cells were washed with PBS containing 0.1% BSA and stained with PI buffer (40 μg/mL containing 100 μg/mL RNase) for 30 min before flow cytometric analysis.

### Colony formation assay

Cells that underwent different drug treatments were plated in 10% FBS medium. After incubation for 10-14 days, the cells were washed with ice-cold PBS, fixed with 20% methanol, and stained with 0.5% crystal violet. The morphology of colonies was imaged under a stereomicroscope. The colony is defined as a cluster of at least 50 cells. The numbers of colonies were counted.

### Sphere formation assay

The sphere formation assay was performed as described previously [26]. To establish sphere cultures, single cells were cultured in 200 μl serum-free DMEM/F12 medium (Gibco) supplemented with 20 ng/ml human recombinant epidermal growth factor (EGF, PeproTech), 20 ng/ml human recombinant basic fibroblast growth factor (bFGF, PeproTech), and B27 (1:50; Gibco). The cells were cultured at a density of 5 × 10^2^ cells/well in ultra-low attachment plates with different treatments for 7 days, and all of the spheres in each well were imaged.

### Transwell migration assay

For this assay, 1 × 10^5^ cells in serum-free medium were seeded in the upper compartment of a Transwell chamber (Corning, NY, USA) with different doses of pimozide. After the cells were incubated with pimozide for 48 h, the migrated cells on the lower membrane were stained with 0.1% crystal violet and then counted.

### Sorting of HCC stem-like cells

HCC stem-like cells were collected for functional assays using SP cells or CD133-positive cells from the MHCC-97L cell line sorted by flow cytometry.

The SP analysis procedures were based on a previously described protocol [27]. Briefly, the cells were trypsinized and suspended in DMEM with 2% FBS and 10 mM HEPES buffer. Next, the cells were stained for 90 min with 5 μg/ml Hoechst 33342 dye (Invitrogen, USA) in the presence or absence of 50 μM verapamil. The incubation was performed with shaking at intervals. After the cells were incubated, they were resuspended in ice-cold PBS containing 2 μg/ml PI. Flow cytometric analysis was performed using a MoFlo XDP cell sorter (Beckman Coulter, Fullerton, CA). The SP gate was defined as the diminished area on the dot plot in the presence of verapamil. The SP MHCC-97L cells were used to evaluate the effects of pimozide.

The CD133-positive population in the MHCC-97L cell line was sorted by flow cytometry using a MoFlo XDP cell sorter (Beckman Coulter, Fullerton, CA). The cells were stained with PE-conjugated anti-human CD133/1 (clone AC133-MAC, Miltenyi Biotec, Auburn, CA, USA). Isotype-matched mouse immunoglobulins served as controls. The CD133-positive cells in the MHCC-97L cell line were used for further functional analysis.

### Western blotting

Equal amounts of protein from each sample were subjected to SDS-PAGE and then transferred to nitrocellulose membranes (Merck Millipore, Billerica MA, USA). The blots were incubated with primary antibodies against GAPDH (Ambion, Austin, TX, USA), p21, Bmi1, Nanog, E-cadherin, N-cadherin, vimentin, phospho-STAT3 (p-STAT3(Ser727) and p-STAT3(Tyr705)), STAT3 (Cell Signaling Technology Corp, Beverly, MA, USA), p27, cyclin D1, c-Myc, Oct3/4 and β-actin (Santa Cruz Biotechnology, Santa Cruz, CA, USA). Antibody binding was detected using an enhanced chemiluminescence kit (Sigma, St. Louis, MO, USA).

### STAT3 luciferase reporter assay

Transient transfections were conducted using Lipofectamine 2000 (Invitrogen, USA) according to the manufacturer's instructions. For the luciferase reporter assay, HEK293T cells were seeded in 24-well plates and transfected with the STAT3 luciferase reporter plasmid STAT3-Luc (pGL3.0, Promega, USA). The cells were collected 48 h after transfection, and the luciferase activities in the cell lysates were determined using the Dual Luciferase Reporter Assay System (Promega, WI, USA). Each transfection was performed in triplicate and repeated at least three times.

### RNA isolation and real-time PCR

RNA was extracted using TRIzol (Invitrogen) according to the manufacturer's protocol and reversely transcribed into cDNA using a Revert Aid First-Strand cDNA Synthesis Kit (Thermo Scientific, USA). The primers are provided in Supplemental Table 1. GAPDH expression was used as an internal control.

### *In vivo* tumorigenicity experiments

MHCC-97L cells were resuspended in PBS, and then 5 × 10^6^ cells were injected subcutaneously into 8-week-old male nude mice (BALB/C, nu/nu). A small tumour formed a week after the tumor cell inoculation, and mice with established disease were divided into cohorts (*n* = 5 per group) with matched tumour burdens. Mice were treated with either 25 mg/kg pimozide in PEG2000 (Sigma, St. Louis, MO) by oral gavage (PO) or a vehicle at the same volume PO. The treatments were administered continuously for 7 days. The tumor volumes and body weights were determined at different times. All studies involving mice were performed according to protocols approved by the Animal Care and Use Committee of The Third Affiliated Hospital of Sun Yat-sen University.

### Statistical analysis

The data, which are presented as the mean ± SD, were analysed using GraphPad Prism 6.0 (GraphPad Software, Inc.). Student's t-test was used to compare the differences between two groups. The level of significance was set at *p* < 0.05.

## SUPPLEMENTARY MATERIALS FIGURES AND TABLE


