# Supplementary material for: The neuroleptic drug pimozide inhibits stem-like cell maintenance and tumorigenicity in hepatocellular carcinoma
Source: Oncotarget. 2015 May 27;8(11):17593–609. doi: 10.18632/oncotarget.4307 (PMC5392272; doi:10.18632/oncotarget.4307)
Supplement: Supplementary file 1 [file oncotarget-08-17593-s001.pdf]

The neuroleptic drug pimozide inhibits stem-like cell maintenance and tumorigenicity in hepatocellular carcinoma

Supplementary Material

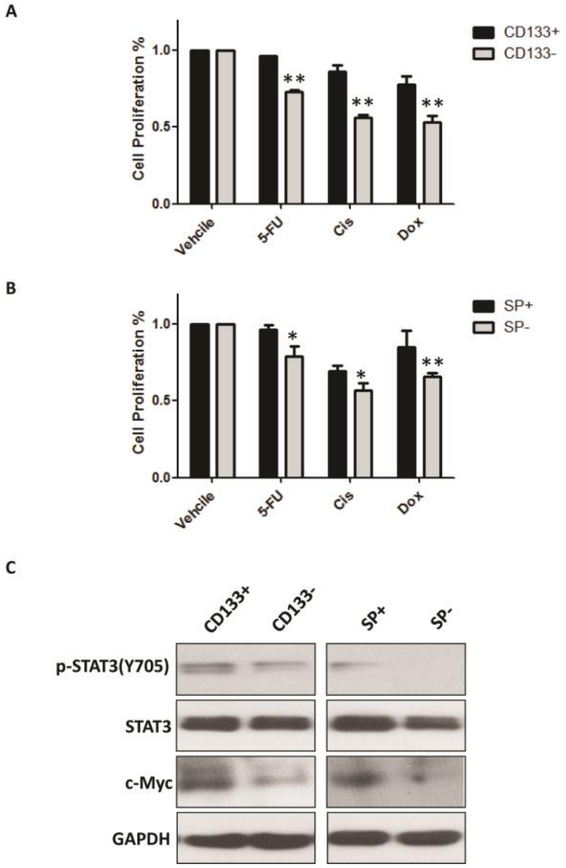

Supplemental Figure 1: CD133-positive cells or SP cells from MHCC-97L cells are respectively resistant to chemotherapy with intracellular high STAT3 activation. CD133-positive cells or SP cells from MHCC-97L cells were incubated with the indicated doses of chemotherapy for 24h using MTT assay. The results showed that CD133-positive cells **(A)** or SP+ cells (B) from MHCC-97L were respectively resistant to fluorouracil (5-FU) (50μM), cisplatin (Cis) (5 μg/ml) and doxorubicin (Dox) (1 μg/ml), compared with their control CD133 negative cells or SP- cells. Relative representative figures are shown. The data are summarized from three independent experiments, *$p < 0.05$, **$p < 0.01$. (C) Western blot analysis of the expression of p-STAT3(Y705), STAT3 and c-Myc in CD133-**positive** cells or SP cells from MHCC-97L cells, compared with their control cells.

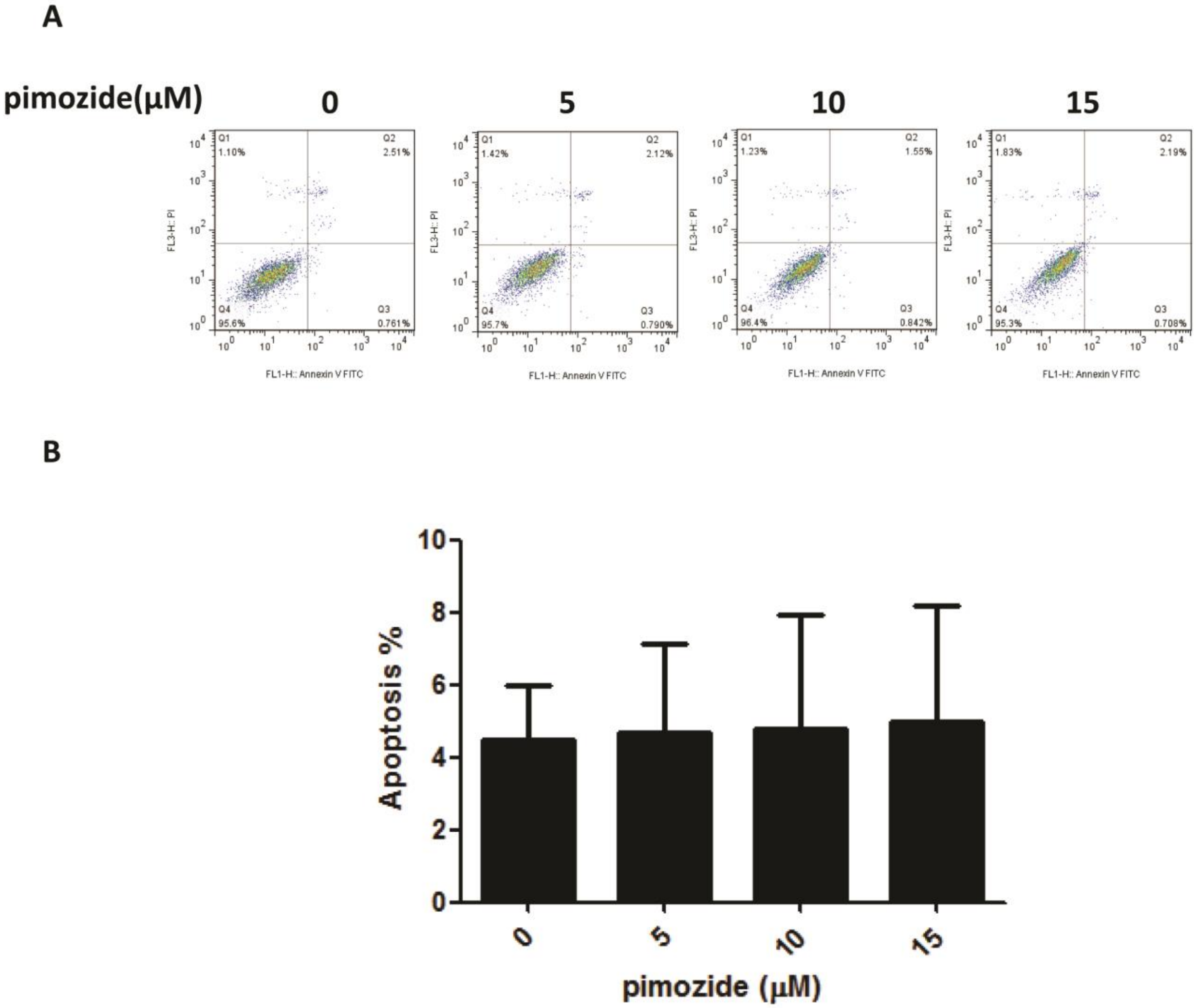

Supplemental Figure 2: Pimozide does not induce obvious apoptosis in MHCC-97L cells. MHCC-97L cells were treated with pimozide at different concentrations for 24h. (A) Apoptotic cells were measured by Annexin V/PI staining. (B) Data summarized three independent experiments, *p<0.05, **p<0.01, compared to control.

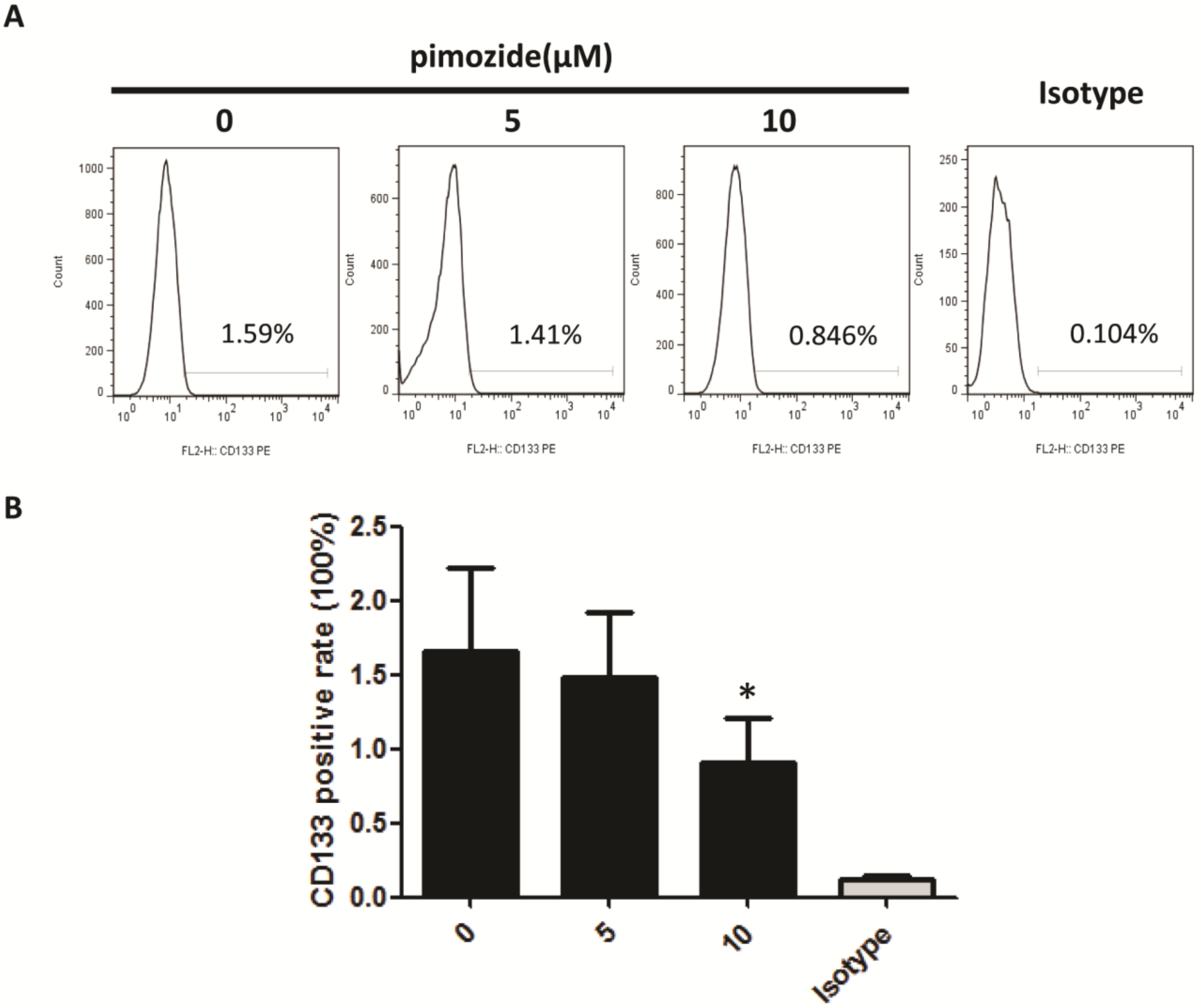

Supplemental Figure 3: Pimozide reduces CD133 positive populations in MHCC-97L cells. MHCC-97L cells were treated with pimozide at different concentrations for 48h. (A) CD133 positive rate was measured by flow cytometry. (B) Data summarized three independent experiments, *p<0.05, **p<0.01, compared to control.

Supplemental Table I: The primers for qPCR

Name	Sense sequence(5'-3')	Antisense sequence(5'-3')
GAPDH	TCCCACTCTTCCACCTTCGATGC	GGGTCTGGGATGGAAATTGTGAG
MCL1	GGCAGGATTGTGACTCTC	CTCCTACTCCAGCAACAC
BCL-xL	GCTGGTGGTTGACTTTCTCTC	GGTCTCCATCTCCGATTCAGT
c-Myc	AGGAACAAGAAGATGAGGAAGA	CTGCGTAGTTGTGCTGATG
Bmi1	GTTCACAAGACCAGACCACTAC	GGCAGCATCAGCAGAAGGA
Nanog	AACTCTCCAACATCCTGAACCT	CTGCGTCACACCATTGCTATT
Oct4	GACAGGGGGAGGGGAGGAGCTAGG	CTTCCCTCCAACCAGTTGCCCCAAAC
